# Genomic content of chemosensory genes correlates with host range in wood-boring beetles (*Dendroctonus ponderosae*, *Agrilus planipennis*, and *Anoplophora glabripennis*)

**DOI:** 10.1186/s12864-019-6054-x

**Published:** 2019-09-02

**Authors:** Martin N. Andersson, Christopher I. Keeling, Robert F. Mitchell

**Affiliations:** 1 Department of Biology, Lund University, Sölvegatan 37, SE-223 62 Lund, Sweden; 2 Laurentian Forestry Centre, Canadian Forest Service, Natural Resources Canada, 1055 rue du P.E.P.S, Stn. Sainte-Foy, P.O. Box 10380, Québec, QC G1V 4C7 Canada; 3 Département de biochimie, de microbiologie et de bio-informatique, Faculté des sciences et de génie, Université Laval, pavillon Alexandre-Vachon, 1045, av. de la Médecine, local 3428, Québec, QC G1V 0A6 Canada; 4 Department of Biology, University of Wisconsin Oshkosh, Oshkosh, WI 54901 USA

**Keywords:** Coleoptera, Odorant receptor, Ionotropic receptor, Gustatory receptor, Odorant binding protein, Chemosensory protein, Sensory neuron membrane protein, Host specificity, Pest insect

## Abstract

**Background:**

Olfaction and gustation underlie behaviors that are crucial for insect fitness, such as host and mate selection. The detection of semiochemicals is mediated via proteins from large and rapidly evolving chemosensory gene families; however, the links between a species’ ecology and the diversification of these genes remain poorly understood. Hence, we annotated the chemosensory genes from genomes of select wood-boring coleopterans, and compared the gene repertoires from stenophagous species with those from polyphagous species.

**Results:**

We annotated 86 odorant receptors (ORs), 60 gustatory receptors (GRs), 57 ionotropic receptors (IRs), 4 sensory neuron membrane proteins (SNMPs), 36 odorant binding proteins (OBPs), and 11 chemosensory proteins (CSPs) in the mountain pine beetle (*Dendroctonus ponderosae*), and 47 ORs, 30 GRs, 31 IRs, 4 SNMPs, 12 OBPs, and 14 CSPs in the emerald ash borer (*Agrilus planipennis*). Four SNMPs and 17 CSPs were annotated in the polyphagous wood-borer *Anoplophora glabripennis.* The gene repertoires in the stenophagous *D. ponderosae* and *A. planipennis* are reduced compared with those in the polyphagous *A. glabripennis* and *T. castaneum*, which is largely manifested through small gene lineage expansions and entire lineage losses. Alternative splicing of GR genes was limited in *D. ponderosae* and apparently absent in *A. planipennis*, which also seems to have lost one carbon dioxide receptor (GR1). *A. planipennis* has two SNMPs, which are related to SNMP3 in *T. castaneum. D. ponderosae* has two alternatively spliced OBP genes, a novel OBP “tetramer”, and as many as eleven IR75 members*.* Simple orthology was generally rare in beetles; however, we found one clade with orthologues of putative bitter-taste GRs (named the “GR215 clade”), and conservation of IR60a from *Drosophila melanogaster.*

**Conclusions:**

Our genome annotations represent important quantitative and qualitative improvements of the original datasets derived from transcriptomes of *D. ponderosae* and *A. planipennis*, facilitating evolutionary analysis of chemosensory genes in the Coleoptera where only a few genomes were previously annotated. Our analysis suggests a correlation between chemosensory gene content and host specificity in beetles. Future studies should include additional species to consolidate this correlation, and functionally characterize identified proteins as an important step towards improved control of these pests.

**Electronic supplementary material:**

The online version of this article (10.1186/s12864-019-6054-x) contains supplementary material, which is available to authorized users.

## Background

Interpreting chemical information in the environment is of paramount importance for the fitness of animals. In insects, the chemical senses –olfaction and taste– underlie the ability to find mates, food, and oviposition sites, and to avoid harmful situations and non-host habitats [1]. The chemosensory multi-gene families are typically the largest gene families of insect genomes, emphasizing the key role of olfaction and taste in insect ecology. In addition, understanding the ‘birth-and-death’ evolution of these genes is needed to gain insight into the mechanisms underlying ecological specialization, evolutionary divergence, and speciation [2, 3], and it may also reveal molecular targets that can be manipulated for insect control, such as pheromone receptors [4]. How the ecology and life history traits of different species relate to the evolution of their chemosensory genes is, however, still an open question. To shed further light on this relationship, we targeted four different beetles (Coleoptera) that are keystone species in forest ecosystems and also serious pests, including the mountain pine beetle *Dendroctonus ponderosae* Hopkins (Curculionidae), the emerald ash borer *Agrilus planipennis* Fairmaire (Buprestidae), and the Asian longhorn beetle *Anoplophora glabripennis* Motschulsky (Cerambycidae). The aim was to investigate how their host breadth (stenophagy vs. polyphagy) may correlate with the diversification of genomic repertoires of chemosensory genes, and at the same time reveal genes that can be targeted for improved control of these forest pests [4].

Most insect chemosensory genes encode membrane-bound receptors from three divergent gene families, which generally are expressed in primary sensory neurons. The odorant receptors (ORs) represent the principal means of sensing volatile chemicals from a distance, including pheromones and odorants from plants and microbes [5]. The receptor genes are mainly expressed in the olfactory sensory neurons (OSNs) of the insect antennae and maxillary palps. ORs are seven-transmembrane domain proteins with an intracellular N-terminus, and are unrelated to vertebrate ORs, which are G-protein coupled receptors [6–10]. In most cases, a single ligand-specific OR gene is expressed in each OSN together with the olfactory receptor co-receptor *Orco*, which is present in all insect genomes except for those of the most basal lineages [11, 12]. The OR and Orco proteins form a heterotetrameric receptor complex [13], where Orco is required for the formation of a ligand-gated cation channel, and hence odor-evoked responses in OSNs [14–16]. The more ancient and even more diverse family of gustatory receptors (GRs) [17, 18] belongs to the same superfamily as the ORs [19, 20]. These genes are expressed in a variety of tissues, and are mainly involved in contact chemoreception of e.g., sugar and bitter compounds, but also in the sensing of carbon dioxide [21, 22]. The third family encodes the ionotropic receptors (IRs), which are related to the conserved ionotropic glutamate receptors (iGluRs), but they have atypical binding domains and are expressed in a combinatorial fashion in sensory neurons [23, 24]. The conserved ‘antennal’ IRs are involved in olfaction [23, 25, 26], but also in the sensing of humidity, salt, and temperature [27–29]. Members of the ‘divergent’ group of IRs, which is highly variable within and between species, have been assigned a role in taste [25, 30].

Proteins from additional gene families play various other roles in insect chemosensation. Genes encoding the sensory neuron membrane proteins (SNMPs), which are related to scavenger proteins of the CD36 family, are expressed in certain OSNs that express OR genes [31]. Two representatives (*Snmp1* and *Snmp2* genes) are found in most insect genomes [32], and a third representative (*Snmp3*) has been identified in Coleoptera [33]. SNMP1 is important for pheromone responses in *Drosophila melanogaster* and moths [31, 34–36], whereas potential roles of SNMP2 and 3 in chemosensation remain unknown. The lymph of chemosensory sensilla also contains abundant small soluble odorant binding proteins (OBPs) [37, 38], which bind, solubilize, and transport hydrophobic odor molecules, and may also serve as a form of gain control, buffering changes in the odor environment [39–42]. Finally, members of the chemosensory protein (CSP) family, of which some are abundant in the sensillum lymph, have also been shown to bind odorants [43, 44], and may thus have similar roles as the OBPs. However, CSP genes are expressed also in non-chemosensory tissues and some have non-sensory functions [37, 45, 46].

The links between chemosensory gene evolution and ecological specialization are poorly understood, although it has been hypothesized that traits, such as host breadth and social behavior, may correlate with the diversification of these gene families [5]. Analyses of insect genomes are crucial in addressing this question, because studies of antennal transcriptomes, which is a more common approach [47], generally miss a large proportion of the genes present in the genome (cf. [48, 49]). While chemosensory genes from the Diptera, Hymenoptera, and Lepidoptera have been more thoroughly compared in this regard [50–52], the largest order of insects, Coleoptera, remains poorly investigated. Apart from the OR genes, which were recently analyzed across ten coleopteran genomes [53], full genomic repertoires of GR, IR and OBP genes have only been identified in three species: the flour beetle *Tribolium castaneum* [54, 55], the Asian longhorn beetle *A. glabripennis* [56], and the Colorado potato beetle *Leptinotarsa decemlineata* [49]. The CSP and SNMP genes have so far only been reported from the genome of *T. castaneum* [33, 57].

To test the hypothesis that variation in host range may be reflected in chemosensory gene repertoires, we annotated these genes in the genomes of two stenophagous wood-boring and phloem-feeding pests of the Coleoptera: the mountain pine beetle *D. ponderosae*, and the emerald ash borer *A. planipennis*. Proteins encoded by these genes were phylogenetically analyzed with those from the polyphagous species *T. castaneum* and *A. glabripennis*, with the latter species being particularly relevant because it also exhibits a wood-boring lifestyle, and it has been recorded from > 100 host plants [58]. In contrast, *T. castaneum* feeds on a large variety of seeds and other dried foods. *D. ponderosae* is a devastating North American bark beetle, able to kill a number of pine (*Pinus*) species over landscape-scales through mass attacks coordinated by an aggregation pheromone [59, 60]. As a result of warming temperatures, current outbreaks have caused unprecedented economic loss, and turned North American forests into sources of carbon release [61]. *A. planipennis* is native to Eastern Asia, but has become an invasive pest in North America, threatening the existence of several ash (*Fraxinus*) species [62]. Compared to *D. ponderosae*, the chemical ecology of this species is less understood, although odor-mediated attraction to hosts and conspecifics has been shown [63–65]. Previous studies of the antennal transcriptomes of these wood-borers identified initial sets of chemosensory genes in *D. ponderosae* [66] and *A. planipennis* [67]. However, subsequent evolutionary and functional analysis have been hampered because both datasets are incomplete (especially for *A. planipennis*), i.e., they include a comparatively small number of identified chemosensory genes, lack expected orthologues, and include many partial gene models. A recent study included the genomic annotations of the ORs of these two species in a large-scale analysis [53], but we here reanalyze them alongside the other chemosensory genes in the context of host specificity. Our analysis suggests a correlation between the genomic content of chemosensory genes and host range in beetles. The annotations presented here are an important contribution to the pool of known chemosensory genes within the Coleoptera, facilitating future evolutionary and functional studies, which in turn may lead to more efficient and sustainable pest control tactics.

## Results

### Improved sets of chemosensory genes in *D. ponderosae* and *A. planipennis*

A previous study of the antennal transcriptome of *D. ponderosae* (“Dpon”) identified a total of 111 chemosensory genes, including 49 ORs, 2 GRs, 15 IRs, 3 SNMPs, 31 OBPs, and 11 CSPs [66]. Our present study of the genome yielded a total of 254 chemosensory genes (Additional file 1: Table S1), of which 153 gene models were not identified in the previous transcriptome. New genes were identified in all gene families, with the largest increase observed for the GRs, followed by the ORs and IRs (details in sections below). Several of the original partial transcript sequences were extended (often to full length), and errors due to previously unnoticed frameshifts or introns on several original transcript models were corrected (Additional file 2: Table S2). Ten of the original gene models were discarded: one OR gene (previous *DponOr45*) was the result of a transcript chimera; one IR gene (previous *DponIr56e.1*) showed no homology to insect IRs; two IR gene fragments (previous *DponIr21a.2* and *56e.2*) were dropped because they were revealed to belong to the same genes as two other previously reported partial IR genes; one IR gene (previous *DponIr93a.2*), four OBP genes (previous *DponObp17*, *20*, *24*, *32*), and one CSP gene (previous *DponCsp5*) were assembly isoforms or alleles of other genes (Additional file 2: Table S2). Only one gene model (*DponOr21*) that was complete in the transcriptome study was incomplete in the genome assemblies; hence the original model was retained.

In *A. planipennis* (“Apla”), 24 chemosensory genes (2 ORs, 2 GRs, 6 IRs, 1 SNMP, 9 OBPs, and 4 CSPs) were previously identified from an antennal transcriptome [67]. Here, we annotated a total of 137 chemosensory genes from its genome, of which 118 annotations are novel compared to previous transcriptome work. Several of the original models were revised (Additional file 2: Table S2) or discarded: two OBP genes (previous *AplaObp4* and *AplaObp8*) were discarded because they were assembly isoforms; two IR genes were identified as separate fragments of *AplaIr25a* (isotig01857-ApIR and G3QO8C008JMTAX_ ApIr); and both existing short AplaGR gene models were found to lack homology to insect GRs. Several of the original gene models were renamed, especially in *D. ponderosae* (most ORs, some IRs, and two SNMPs; see also [53]) to follow established nomenclature for genomic annotations of these gene families (see Methods section for details and Additional file 2: Table S2 for correspondence with original names).

### Odorant receptors

A total of 86 OR genes, including *Orco* and 7 putative pseudogenes, were annotated in the genome of *D. ponderosae*, of which 63 OR genes were completed to full-length. Except for DponOR53INT (195 amino acids), all partial but putatively functional DponORs are above 350 amino acids in length, with the majority only missing a short N-terminal exon (named A1) that could not be confidently identified due to absence of transcriptomic support. As has been observed with the OR genes in other insect genomes, a large proportion of the genes in both species occur in tandem arrays on scaffolds (Additional file 1: Table S1). Although alternative splicing is uncommon in ORs, two of the DponOR genes were regarded to each encode two alternative splice variants (named *DponOr2a/b* and *DponOr36a/b*) with mutually exclusive N-terminal A1-A2 exons assembled consecutively with seemingly shared C-terminal B-E exons. The *A. planipennis* genome contains 47 OR genes, including *Orco* and one pseudogene. In this species, 31 of the ORs were completed to full-length models, with partial OR genes encoding protein sequences between 174 and 393 amino acids. In both species, the number of introns in full-length OR genes varies between four and seven, whereas *Orco* is interrupted by ten introns in both species (Additional file 1: Table S1; see also [53]). Both *D. ponderosae* and *A. planipennis* have fewer putatively functional OR genes and pseudogenes compared to other species considered polyphagous (Table 1).

**Table 1:** Numbers of putatively functional proteins and pseudogenes (in brackets) in five beetle species in which the majority of the chemosensory gene families have been annotated from their genomes

	ORs	GRs	IRs	SNMPs	OBPs	CSPs
*Dendroctonus ponderosae*	**79 (7)**	**59 (1)**	**55 (2)**	**4**	**36**	**11**
*Agrilus planipennis*	**46 (1)**	**30**	**30 (1)**	**4**	**12**	**14**
*Anoplophora glabripennis*	121 (11)	190 (44)	63 (9)	**4**	60 (1)	**17**
*Tribolium castaneum*	270 (68)	219 (26)	71 (9)	6	50	20
*Leptinotarsa decemlineata*	76 (4)	144 (3)	27	N/A	58 (1)	N/A

Numbers in bold indicate annotations in the present study

OR – odorant receptor, GR – gustatory receptor, IR – ionotropic receptor, SNMP – sensory neuron membrane protein, OBP – odorant binding protein, CSP – chemosensory protein

**Fig. 1:**
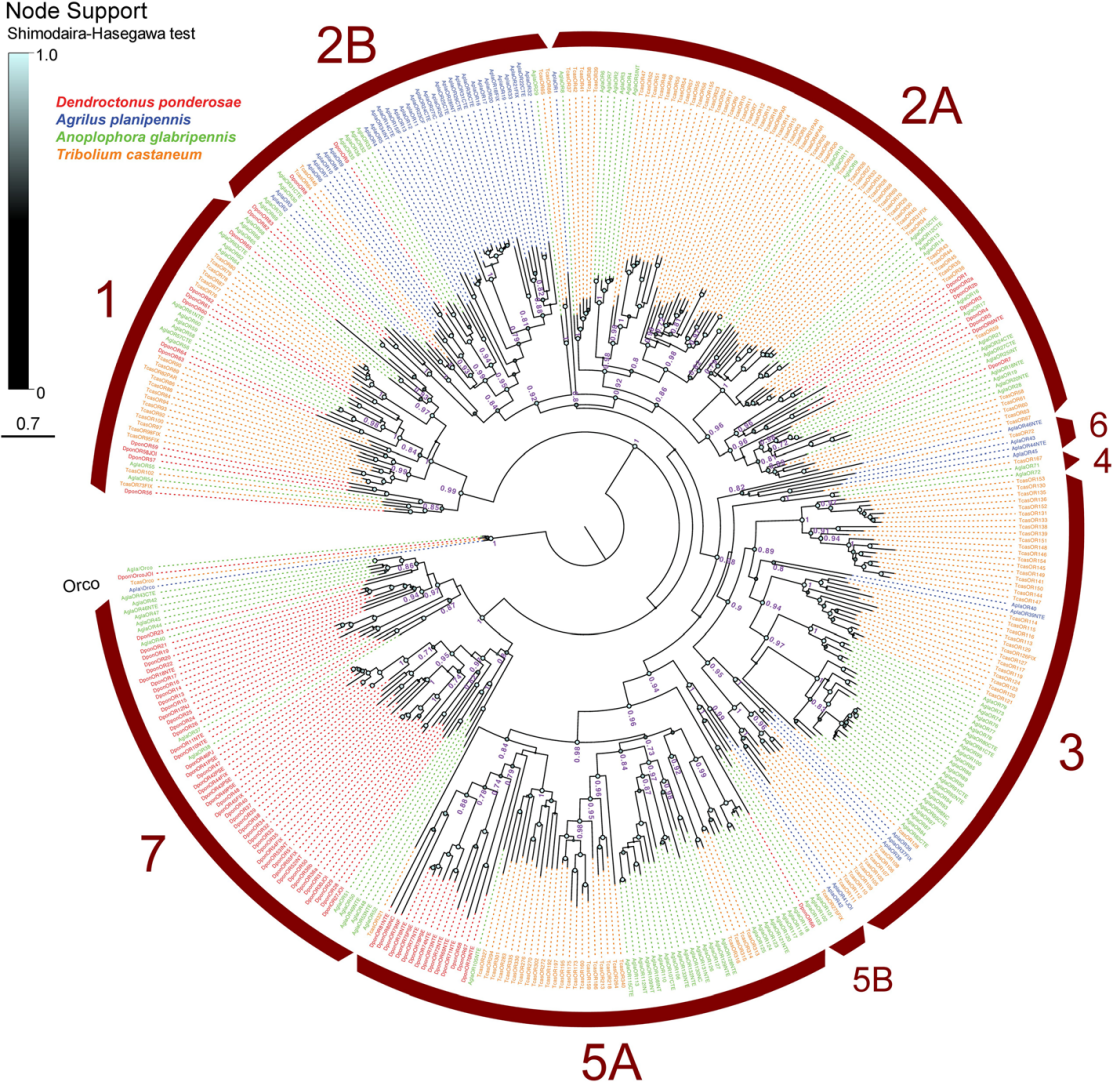
Phylogenetic tree of the odorant receptor (OR) family. The receptor sequences included were from *Dendroctonus ponderosae* (Dpon, red), *Agrilus planipennis* (Apla, blue), *Anoplophora glabripennis* (Agla, green), and *Tribolium castaneum* (Tcas, orange). The tree is based on a MAFFT alignment, constructed using PhyML, and rooted with the conserved lineage of Orco proteins. Numbers at nodes indicate aLRT (approximate Likelihood Ratio Test) SH (Shimodaira-Hasegawa)-like branch support, calculated using PhyML. For clarity, exact support values are only shown for major branches and if > 0.7, whereas support for all branches are indicated by the colored circles; support increases with the size and brightness of the circles. The red arcs indicate the nine major coleopteran OR groups as defined in [53]. To reduce tree size, the massively expanded *T. castaneum-*specific OR-lineages in former OR groups 5 and 6 [54] are here represented by 10 ORs each, which together with the former OR group 4 were recently combined into subfamily 5A [53]. The sources of sequence data and explanation of receptor suffixes are detailed in the Methods section

The DponORs and AplaORs were recently included in phylogenetic analyses that covered ORs from ten coleopteran genomes, and which allowed for classification and revision of nine higher-order monophyletic OR subfamilies (designated as Groups 1, 2A, 2B, 3, 4, 5A, 5B, 6, and 7) across the Coleoptera [53]. Several of these groups had been recognized also in earlier studies [54, 66]. In this study, the phylogenetic analysis was restricted to two additional species (*T. castaneum* “Tcas” and *A. glabripennis* “Agla”), and we can here afford to present and discuss the results for these particular species in more detail. Our phylogeny (Fig. 1) shows that the distribution of ORs among the nine major coleopteran OR subfamilies is species-dependent. The majority of DponORs belong to Group 7, followed by Groups 5A, 1, 2A, and 2B. In contrast, most AplaORs are found within Group 2B, followed by Groups 3, 6, 5B, and 2A. Furthermore, *D. ponderosae* appears to have lost ORs in Groups 3, 4, 5B, and 6, whereas *A. planipennis* lacks ORs in Groups 1, 4, 5A, and 7. These different OR distribution patterns are also distinct from those in *T. castaneum* and *A. glabripennis*, which in turn also are different from each other*.* In *D. ponderosae,* the largest species-specific radiation contained 30 ORs (DponOR27–55 including the putatively alternatively spliced DponOR36a/b in Group 7), whereas the largest expansion in *A. planipennis* contained 18 ORs (AplaOR16–33 in Group 2B). Well-supported orthologous relationships were only found for AglaOR55/DponOR57–59, AglaOR38/DponOR10–11, and TcasOR73FIX/DponOR56.

### Gustatory receptors

In *D. ponderosae*, we annotated 60 GR transcripts (including 57 full-length models and one pseudogene) that are encoded by 49 genes. Seven of these genes were regarded to exhibit alternative splicing, each producing either two or four splice variants (Additional file 1: Table S1). Most splice variants are encoded by genes with two exons, and they share the C-terminal exon, but have a unique N-terminal exon. One of the alternatively spliced genes, *DponGr38a-d,* has three exons, of which the N-terminal exon is unique and the two C-terminal exons are shared. In *A. planipennis*, 30 GR genes were revealed (22 full-length models), with no evidence of alternative splicing. The putative receptors for carbon dioxide and sugars contain several introns, whereas the majority of the remaining putative bitter-taste GR genes contain only one or two introns in both species. However, several of these GRs, especially in *D. ponderosae*, contained one to four additional introns (Additional file 1: Table S1). As with ORs, *D. ponderosae* and *A. planipennis* presented fewer putatively functional GR genes and pseudogenes compared to other species considered polyphagous (Table 1).

**Fig. 2:**
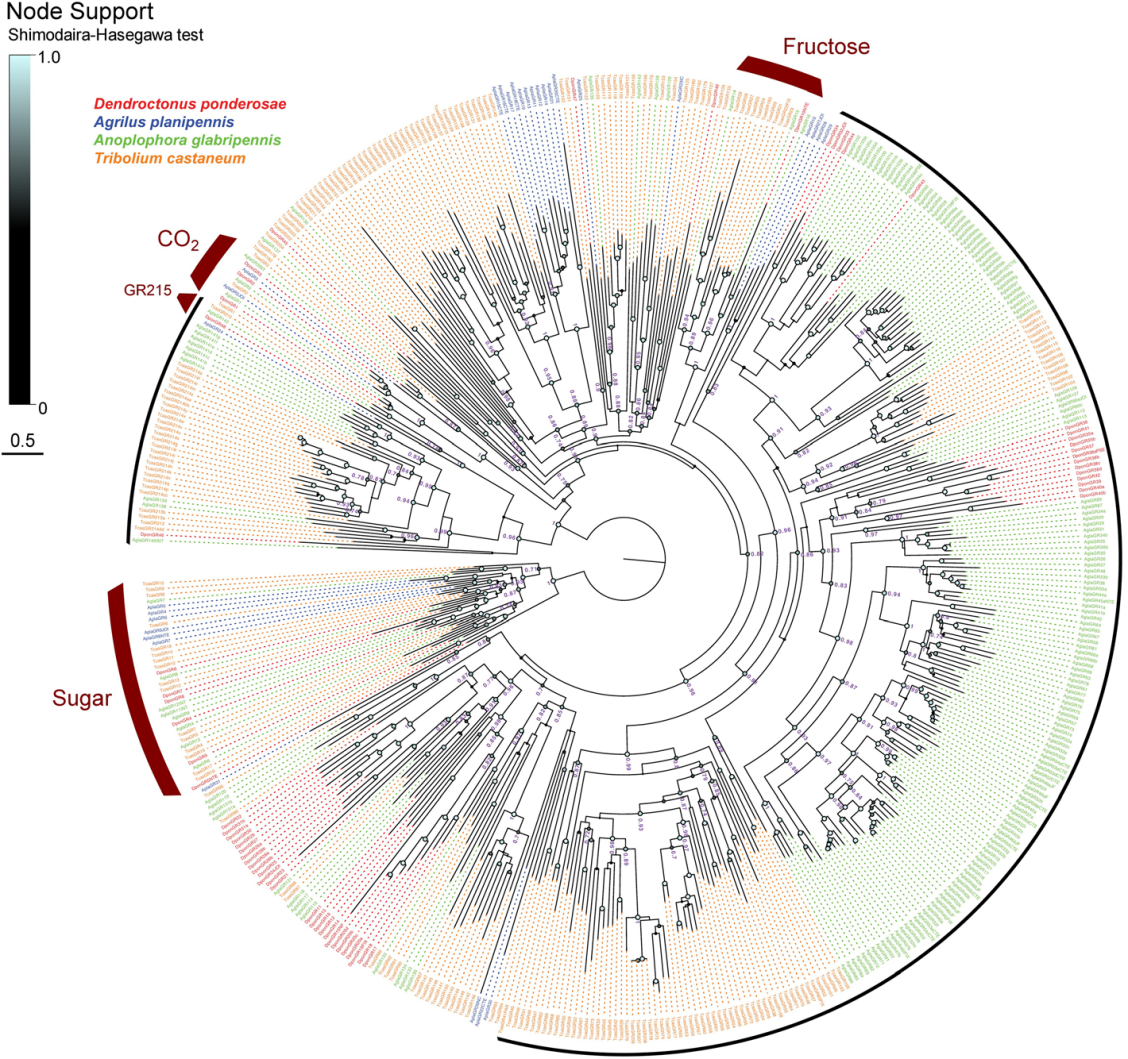
Phylogenetic tree of the gustatory receptor (GR) family. The receptor sequences included were from *Dendroctonus ponderosae* (Dpon, red), *Agrilus planipennis* (Apla, blue), *Anoplophora glabripennis* (Agla, green), and *Tribolium castaneum* (Tcas, orange). The tree is based on a MAFFT alignment, constructed using FastTree, and rooted with the conserved lineage of putative sugar receptors. Numbers at nodes are local support values, calculated using the Shimodaira-Hasegawa (SH) test implemented in FastTree. For clarity, exact SH values are only shown for major branches and if > 0.7, whereas SH values for all branches are indicated by the colored circles; support increases with the size and brightness of the circles. Well established GR clades with high support across all four beetle species are indicated by thick red arcs; thin black arcs indicate highly supported clades with species-specific differences in the extent of GR lineage-expansion. The sources of sequence data and explanation of receptor suffixes are detailed in the Methods section

The Dpon and AplaGRs were phylogenetically analyzed together with the GRs from *A. glabripennis* and *T. castaneum,* showing that the three conserved GRs for carbon dioxide (GR1–3) are present in *D. ponderosae*, whereas no evidence of GR1 was recovered from the genome assembly of *A. planipennis*, nor from the available raw sequence reads (accession: SRR1174015–SRR1174018; Fig. 2). In addition, both species have six GRs (GR4–9) that grouped within the clade of conserved sugar receptors. Whereas DponGR4, DponGR6, and DponGR9 appear orthologuous to AglaGR4, AglaGR8, and AglaGR6, respectively, no simple orthologuous relationships are evident for the other sugar receptors in *D. ponderosae* or for any of these GRs in *A. planipennis.* These two species also have one GR each (GR10) that was placed within the clade of conserved fructose receptors, which is dominated by a lineage expansion in *T. castaneum.* Most of the remaining GRs (putative bitter-taste GRs) of all four species in the analysis grouped in small to large species-specific expansions, which in many cases comprise large suites of alternatively spliced proteins, especially from *T. castaneum* and *A. glabripennis.* Among the putative bitter-taste GRs, only a single clade was represented by one orthologue from each of the four species. This clade was highly supported (Shimodaira-Hasegawa [SH] support value 1.0) and named the “GR215 clade” based on the GR representative from *T. castaneum* (Fig. 2). The GR215 clade is part of a larger and well-supported subfamily that includes one additional DponGR (DponGR46), and large expansions of alternatively spliced proteins from *T. castaneum* and *A. glabripennis.* Finally, it is noteworthy that one of the largest well-supported GR lineages (indicated by the long black arc in Fig. 2), comprising almost half of the GRs in our analysis, was devoid of GR representatives from *A. planipennis*.

### Ionotropic receptors

**Fig. 3:**
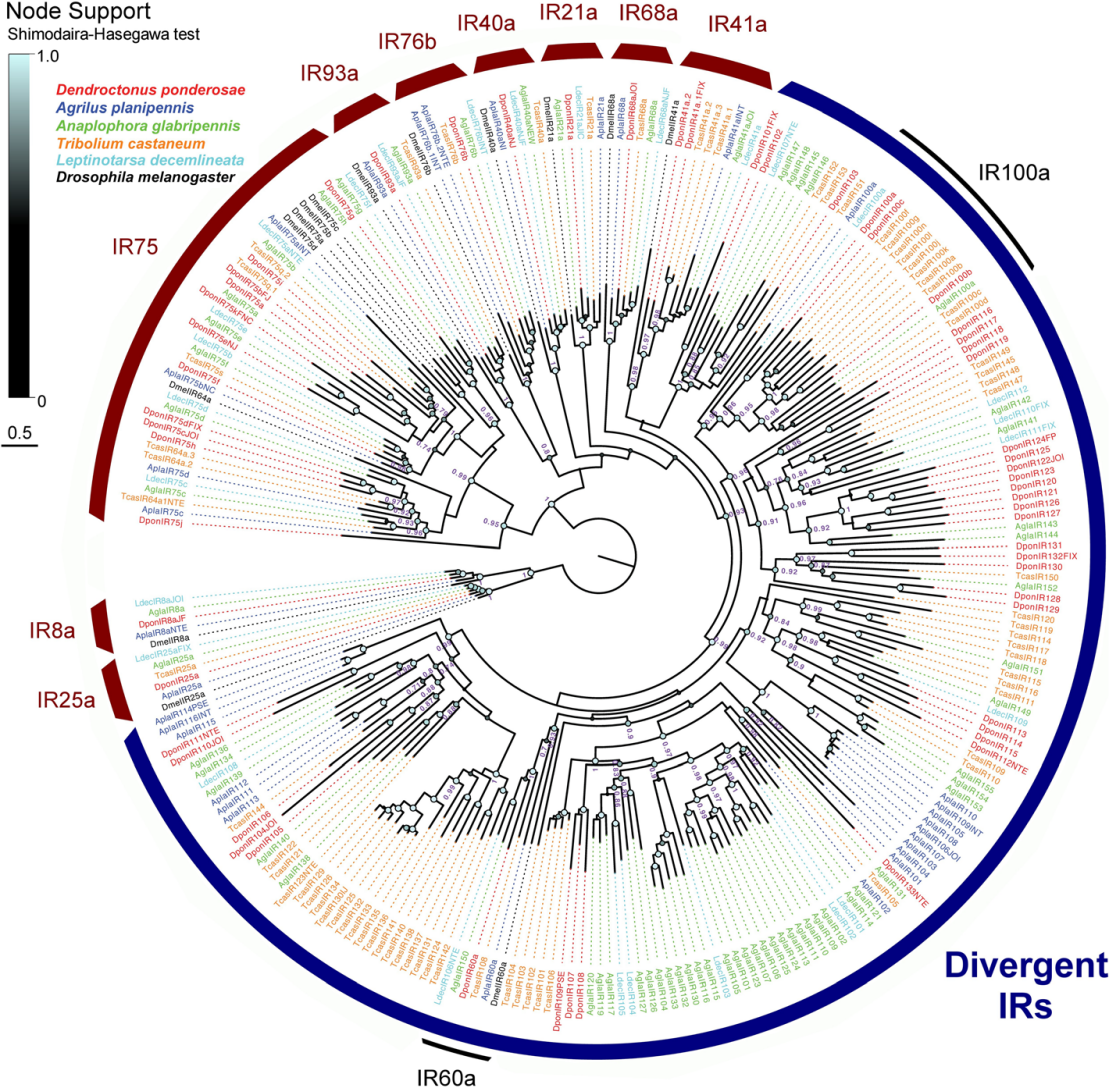
Phylogenetic tree of the ionotropic receptor (IR) family. The receptor sequences included were from *Dendroctonus ponderosae* (Dpon, red), *Agrilus planipennis* (Apla, blue), *Anoplophora glabripennis* (Agla, green), *Tribolium castaneum* (Tcas, orange), *Leptinotarsa decemlineata* (Ldec, cyan), and *Drosophila melanogaster* (Dmel, black; only conserved antennal IR sequences [25]). The tree is based on a MAFFT alignment, constructed using FastTree, and rooted with the conserved lineages of IR8a and IR25a proteins. Numbers at nodes are local support values, calculated using the Shimodaira-Hasegawa (SH) test implemented in FastTree. For clarity, exact SH values are only shown for major branches and if > 0.7, whereas SH values for all branches are indicated by the colored circles; support increases with the size and brightness of the circles. Thick red arcs indicate widely conserved lineages of antennal IRs; the thick blue arc indicates the divergent IRs; thin black arcs indicate highly supported clades (IR60a and IR100a) of conserved IRs that group among the divergent class of IRs. The sources of sequence data and explanation of receptor suffixes are detailed in the Methods section

In *D. ponderosae*, we identified a total of 57 IR genes (51 full-length models), including two pseudogenes. The number of IRs in *A. planipennis* was 31 (22 full-length models), including one pseudogene. Members of the conserved antennal IR8a, IR21a, IR25a, IR40a, IR41a, IR68a, IR76b, and IR93a were identified in both species (Fig. 3). Two paralogues of IR41a and IR76b were annotated in *D. ponderosae* and *A. planipennis*, respectively*.* Furthermore, *D. ponderosae* has 11 IRs that fell within the IR75 clade, which to date is the largest number reported from a beetle genome. In contrast, *A. planipennis* has only four members in this clade. Both species also had members of the IR100a clade, with three receptors found in *D. ponderosae*, and one in *A. planipennis.* Each beetle species (including *L. decemlineata* “Ldec”) also has one IR that grouped with IR60a from *D. melanogaster* (“Dmel”) with high support (0.93), suggesting that this IR is conserved in beetles. Hence, the IRs from this group identified in the present study were named DponIR60a and AplaIR60a, whereas the orthologues from *T. castaneum*, *L. decemlineata*, and *A. glabripennis* retained their original names (TcasIR108, LdecIR106, and AglaIR150). The remaining divergent IRs from *D. ponderosae* and *A. planipennis* generally grouped in species-specific lineage expansions of various sizes, with only a few IRs being individually placed. Whereas the antennal IR genes are known to contain several and often very large introns, the number of introns in the divergent IRs was low (range: 0–3; Additional file 1: Table S1). Again, we observed fewer putatively functional IR genes and pseudogenes in the stenophagous species (Table 1).

### Sensory neuron membrane proteins

**Fig. 4:**
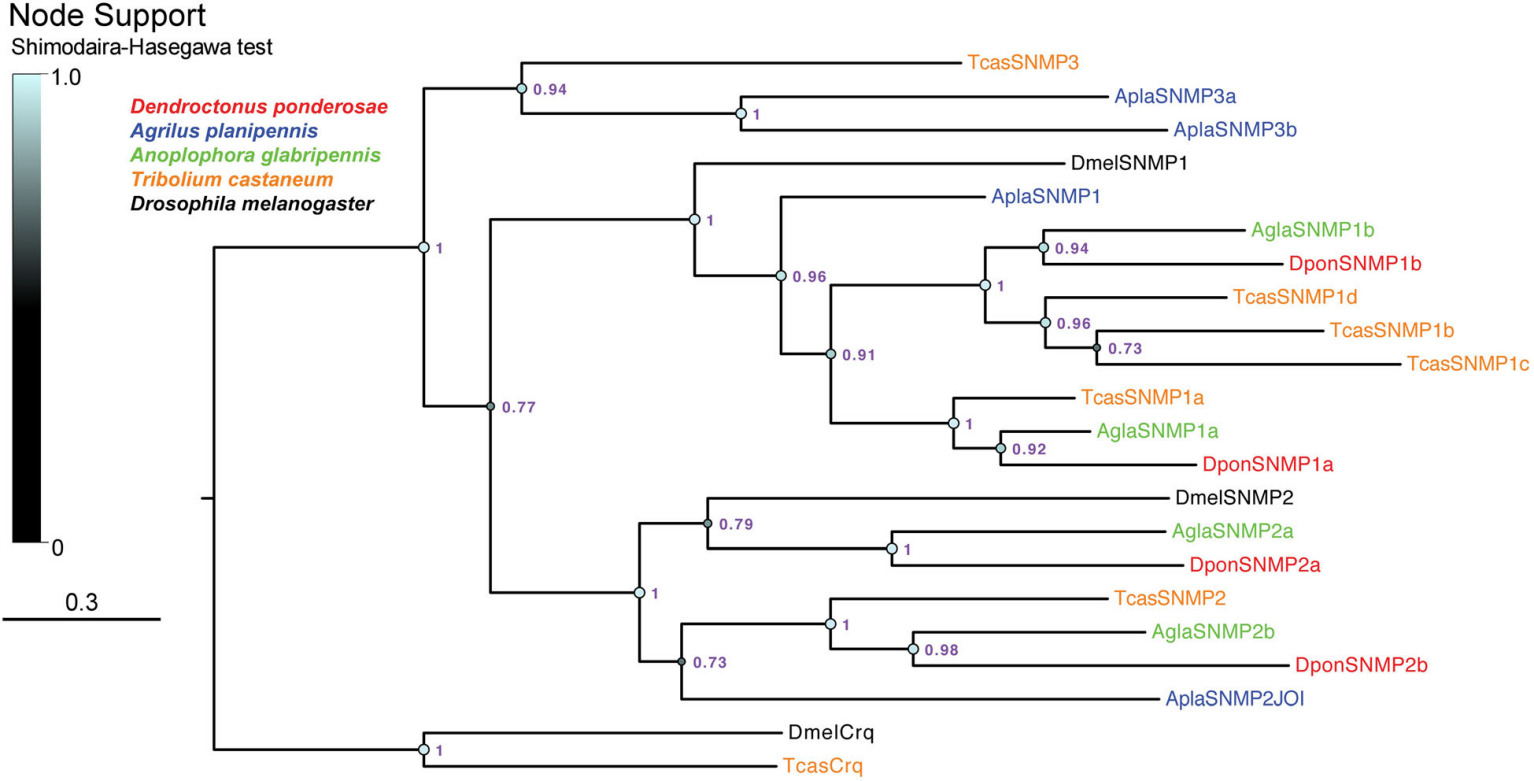
Phylogenetic tree of the sensory neuron membrane protein (SNMP) family. The protein sequences included were from *Dendroctonus ponderosae* (Dpon, red), *Agrilus planipennis* (Apla, blue), *Anoplophora glabripennis* (Agla, green), *Tribolium castaneum* (Tcas, orange), and *Drosophila melanogaster* (Dmel, black). The tree is based on a MAFFT alignment, constructed using FastTree, and rooted with the lineage of Croquemort (Crq) proteins, which are non-SNMP members of the CD36 family. Numbers at nodes are local support values, calculated using the Shimodaira-Hasegawa (SH) test implemented in FastTree. Exact SH values are shown if > 0.7, whereas SH values for all branches are indicated by the colored circles; support increases with the size and brightness of the circles. The sources of sequence data and explanation of receptor suffixes are detailed in the Methods section

We annotated four SNMP genes (all full-length models; Table 1) in each of *D. ponderosae*, *A. planipennis* and *A. glabripennis*. Both *D. ponderosae* and *A. glabripennis* have two members each of SNMP1 and SNMP2, whereas *A. planipennis* only has one member in each of these broadly conserved classes. The two remaining AplaSNMP genes encode proteins related to TcasSNMP3, and were thus named *AplaSnmp3a* and *3b*. The beetle SNMP3 clade was positioned sister to the SNMP1/SNMP2 subfamilies (Fig. 4).

### Odorant binding proteins

**Fig. 5:**
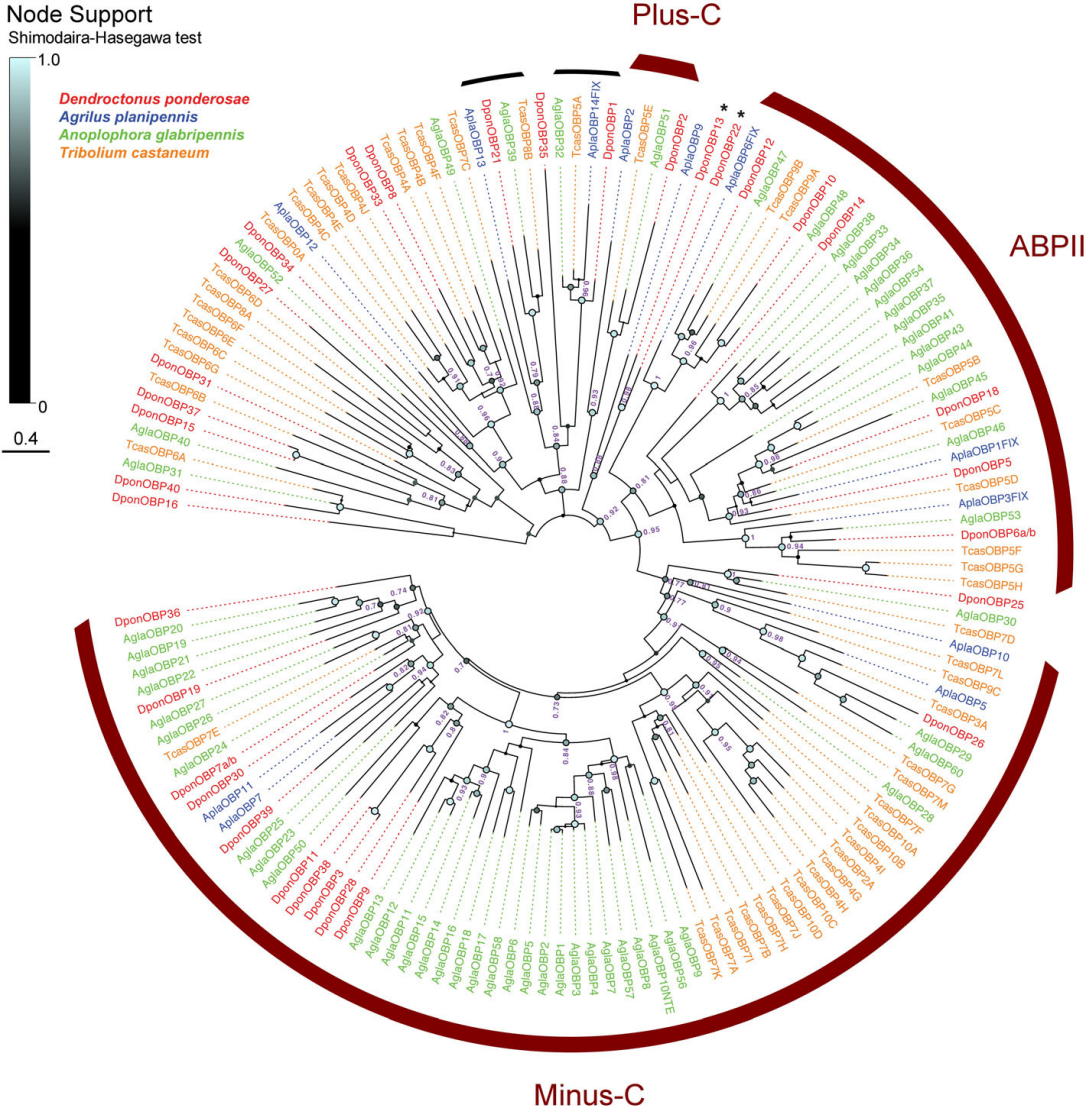
Phylogenetic tree of the odorant binding protein (OBP) family. The protein sequences included were from *Dendroctonus ponderosae* (Dpon, red), *Agrilus planipennis* (Apla, blue), *Anoplophora glabripennis* (Agla, green), and *Tribolium castaneum* (Tcas, orange). The tree is based on a MAFFT alignment, constructed using FastTree, and unrooted. Numbers at nodes are local support values, calculated using the Shimodaira-Hasegawa (SH) test implemented in FastTree. For clarity, exact SH values are only shown for major branches and if > 0.7, whereas SH values for all branches are indicated by the colored circles; support increases with the size and brightness of the circles. The putatively alternatively spliced DponOBP6a/b and DponOBP7a/b are each represented by one splice variant. The OBP “tetramer” DponOBP4 was analyzed separately (Additional file 3). Thick red arcs indicate the clades of Minus-C OBPs, Plus-C OBPs, and the antennal binding protein II (ABPII). Thin black arcs indicate clades of classic OBPs, which include one member from each of the four species in the analysis. Asterisks indicate two Minus-C OBPs from *D. ponderosae*, which in this analysis fell outside the Minus-C clade

Our genome annotations revealed 36 OBPs in *D. ponderosae* and 12 OBPs in *A. planipennis* (all full-length models), which is fewer than in other species considered polyphagous (Table 1)*.* Two of the DponOBP genes (*DponObp37* and *DponObp38*) were exclusive to the male assembly, suggesting that they are located on the neoY chromosome. OBPs are classified into different groups based on the number of conserved cysteine (C) residues and their phylogenetic relationships [37, 68]. The “classic” OBPs share a characteristic pattern of six C residues. Members of the Minus-C class have lost two of these cysteines (generally C2 and C5), whereas the Plus-C OBPs typically have 12 conserved cysteines and a characteristic proline. Finally, one subfamily of the classic OBPs is further classified as “antennal binding protein II” (ABPII), members of which are generally upregulated in the antennae [57]. The inspection of the patterns of C residues and our phylogenetic analysis showed that the genomes of *D. ponderosae* and *A. planipennis* contain one Plus-C member each, similar to other beetles (Fig. 5; Additional file 1: Table S1). Furthermore, 15 DponOBPs and four AplaOBPs presented the 4C pattern that is characteristic of the Minus-C group. However, two of these proteins (DponOBP13 and DponOBP22) did not group within the Minus-C clade in our phylogeny, but were placed with the classic OBPs with intermediate support (SH = 0.88). Seven DponOBPs and three AplaOBPs were placed within the ABPII clade (Fig. 5). In contrast to the polyphagous *A. glabripennis* and *T. castaneum*, no major species-specific lineage expansions were observed in the two stenophagous wood-borers apart from a small expansion of five Minus-C DponOBPs (DponOBP3, 9, 11, 28, 38).

Two of the DponOBP genes (*DponObp6* and *DponObp7*) showed evidence of alternative splicing, supported by transcriptomic data [66, 69]. In both cases, the alternative splicing involves two mutually exclusive N-terminal exons encoding the short signal peptide, which appears to be alternatively combined with a shared C-terminal exon (*DponObp7*; Minus C-group) or six shared exons (*DponObp6*; ABPII group) (Additional file 1: Table S1). Finally, we identified an unusually large OBP in *D. ponderosae* (*DponObp4*), encoding 500 amino acids. Apart from a short N-terminal exon housing the signal peptide, this gene contains four similarly sized exons, each presenting the conserved Minus-C motif. This extraordinary Minus-C “tetramer” model was supported by previous transcriptomic data from this species [66, 69] and two other curculionids: the Yunnan pine shoot beetle (*Tomicus yunnanensis;* accession: GFJU01117056.1) and the red palm weevil (*Rhynchophorus ferrugineus*; GDKA01001723.1), retrieved from the Transcriptome Shotgun Assembly (TSA) collection at NCBI. The first three Minus-C exons of *DponObp4* are separated by short, approx. 60 bp introns, whereas the final exon is separated by a 1.26 kb intron. To investigate how such a large OBP may have originated, we individually aligned the four Minus-C exons of DponOBP4 together with a subset of Minus-C OBPs, i.e., those encompassed under the most recent node shared by the individual DponOBP4 exons. The resulting phylogeny grouped DponOBP4 exons 2 and 3 together with moderate support, while exon 4 was positioned in a sister clade. Exon 5 was widely separated from the other exons but without support (Additional file 3: Figure S1). Inconsistent with this phylogeny, however, exon 2 and 4 shared the highest amino acid identity (41.6%), and a relatively high identity (30.7%) was shared between exon 3 and 5, suggesting that this protein may have originated from a dimer that underwent a duplication of its two major exons.

### Chemosensory proteins

**Fig. 6:**
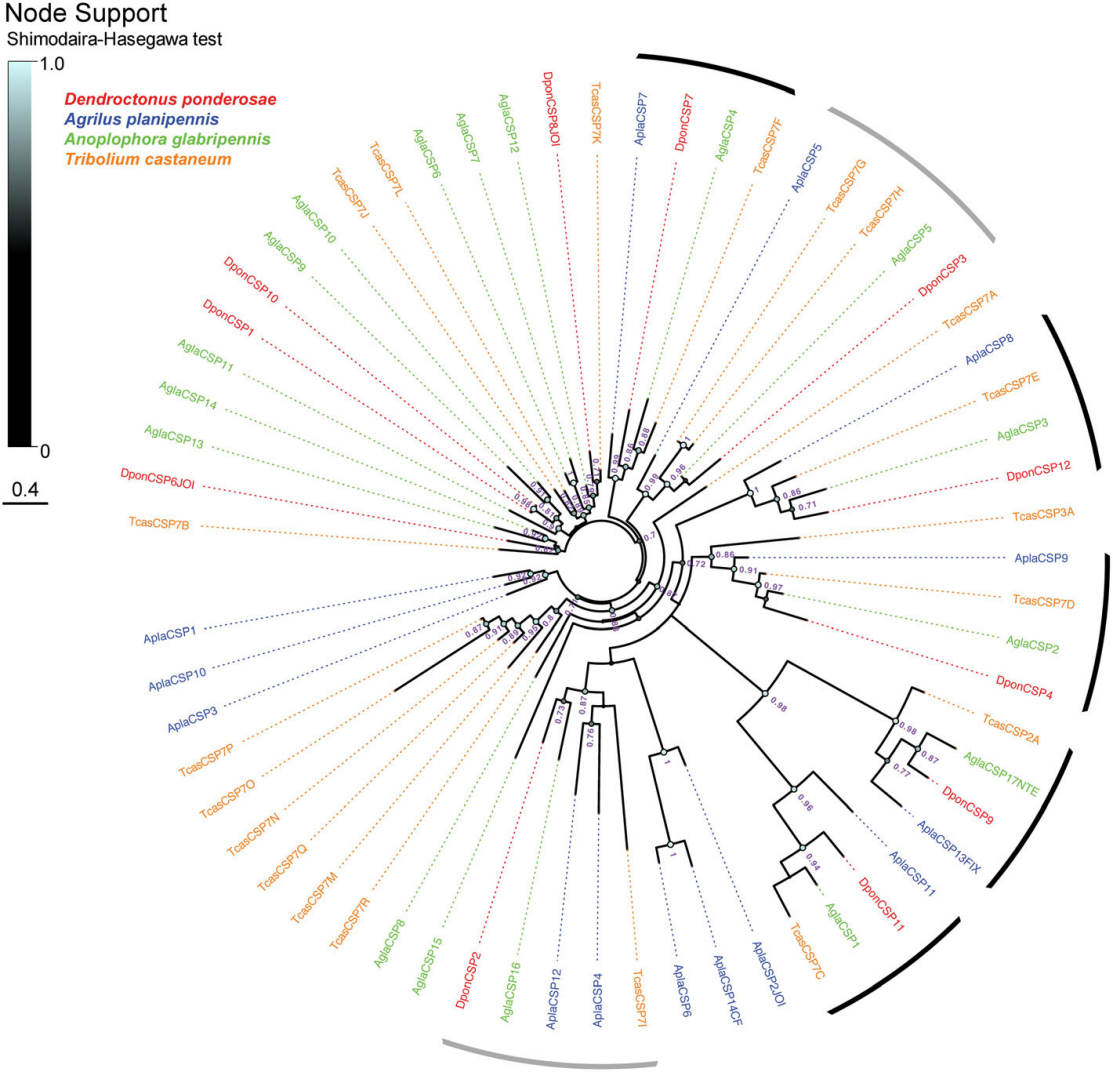
Phylogenetic tree of the chemosensory protein (CSP) family. The protein sequences included were from *Dendroctonus ponderosae* (Dpon, red), *Agrilus planipennis* (Apla, blue), *Anoplophora glabripennis* (Agla, green), and *Tribolium castaneum* (Tcas, orange). The tree is based on a MAFFT alignment, constructed using FastTree, and unrooted. Numbers at nodes are local support values, calculated using the Shimodaira-Hasegawa (SH) test implemented in FastTree. Exact SH values are only shown if > 0.7, whereas SH values for all branches are indicated by the colored circles; support increases with the size and brightness of the circles. Black arcs indicate highly supported clades with a single CSP representative from each of the four species; grey arcs indicate highly supported clades with CSPs from all four species, with one of the species showing evidence of gene duplication. The sources of sequence data and explanation of protein suffixes are detailed in the Methods section

Total numbers of CSP genes were 11 (all full-length models) in *D. ponderosae*, 14 (13 full-length models) in *A. planipennis*, and 17 (16 full-length models) in *A. glabripennis* (Table 1). The majority of the beetle CSP genes are characterized by the presence of a single central intron in splice phase 1, however a few of the DponCSPs have one additional intron (phase 0) close to the N-terminus, with the first exon only coding for the first two amino acids of the protein. Most of the CSP genes within each species were assembled on the same genomic scaffold (Additional file 1: Table S1). The phylogenetic analysis revealed the presence of several highly-supported clades with one or two CSPs from each of the four species, suggesting the existence of several simple orthologous relationships in this gene family (Fig. 6). This includes a conserved clade of four CSPs (DponCSP12, AplaCSP8, AglaCSP3, and TcasCSP7E) with greatly elongated C-terminals and proteins ranging from 251 to 307 amino acids. Species-specific radiations of CSP lineages were rare, but a few smaller ones (comprising 3–6 CSPs) were evident in *T. castaneum* and *A. planipennis.* CSPs from the latter species were missing from a few well-supported clades that contained members from two or three of the other species.

## Discussion

We annotated the chemosensory gene families from the genomes of the two stenophagous wood-borers *D. ponderosae* and *A. planipennis*, and compared their gene repertoires with those in polyphagous species, in particular *T. castaneum* and *A. glabripennis,* with the latter species being a wood-borer recorded from > 100 host trees [58]. Our annotations represent major qualitative and quantitative improvements of the original datasets derived from antennal transcriptomes [66, 67], with a large number of novel sequences identified and several original models extended to full length, corrected or discarded. With the present annotation of the CSPs and SNMPs also in the cerambycid *A. glabripennis*, the six main insect chemosensory gene families have now been identified from genomes of four species of Coleoptera [54, 56], whereas the chemoreceptors and OBPs have been annotated also in a fifth species, *L. decemlineata* [49]*.* Hence, our study facilitates analysis of the molecular evolution of chemosensation in the largest order of insects, Coleoptera, and we can now begin to address questions of how beetle ecology relates to the diversification of these crucial gene families.

### Correlation between chemosensory gene repertoire size and host range

The number of chemosensory genes varies between insect taxa due to different rates of gene gain (via tandem duplication) and loss (via pseudogenization and deletion). This variation is thought to relate to specific lifestyles and adaptations to the environment, and may ultimately result in speciation [2, 3, 5, 51, 70]. We predicted that host-specific insects would have fewer chemosensory genes than polyphagous species. Indeed, the total numbers of such genes in the *Pinus* specialist *D. ponderosae* and the *Fraxinus* specialist *A. planipennis* are lower than in *T. castaneum* and *A. glabripennis* (Table 1)*.* This was consistent across all chemosensory gene families (except the SNMPs), with especially large differences observed among the ORs, GRs, and IRs, followed by the OBPs, especially in *A. planipennis*. The smaller gene numbers in stenophagous species are largely explained by reductions in the extent and number of paralogous radiations in the two specialized wood-borers. Their lower numbers of GRs are at least partly manifested through low frequency of alternative splicing in both *D. ponderosae* and *A. planipennis.* In fact, none of the GR genes in the latter species showed indications of alternative splicing, which is unique among the coleopterans investigated so far. In *D. ponderosae*, seven GR genes are putatively alternatively spliced, but with only two or four splice variants per gene, comprising 18% of the total GRs. Though only 13% of the GRs of *T. castaneum* are splice variants, 30 splice variants are encoded by the single gene *TcasGr214* [54]. In *A. glabripennis* [56], 39% of the GRs are splice variants with up to 17 proteins from a single gene (*AglaGr99*). The same percentage is found in the oligophagous Colorado potato beetle, *L. decemlineata*, which feeds on a variety of solanaceous plants, with up to 13 splice variants encoded by one gene (*LdecGr48*) [49]. However, compared to the two polyphagous species, *L. decemlineata* also has fewer chemoreceptors, especially a highly reduced IR repertoire, and the size of its OR repertoire is similar to that in *D. ponderosae*. *D. ponderosae* and *A. planipennis* also have proportionally fewer pseudogenes than *A. glabripennis* and *T. castaneum*, especially among the GRs. This suggests that the smaller complements of chemoreceptors are due to reduced gene gain via duplication rather than increased gene loss via pseudogenization.

While the observations across these five beetle species suggest a correlation between the number of chemosensory genes and diet breadth, additional species need to be sampled to draw general conclusions, because other ecological factors and the species phylogeny may also play a role. For instance, *A. planipennis* has well-developed eyes, and visual stimuli are important for mate and host attraction [63, 71], which might correlate with reduced reliance on chemosensation. This hypothesis is in line with our results, with this species having the lowest number of chemosensory genes, including to our knowledge the fewest OBPs recorded from a holometabolous insect. Secondly, our sampling included single representatives from four families (Apla: Buprestidae, Tcas: Tenebrionidae, Agla: Cerambycidae, and Dpon: Curculionidae) unevenly distributed across the coleopteran phylogeny, which may have influenced the outcome of the analysis. *A. planipennis* is part of the relatively distant infraorder Elateriformia, whereas the other species belong to the “higher beetles”, Cucujiformia [72]. Indeed, previous antennal transcriptome studies of species from early diverging lepidopteran lineages and caddisflies (sister taxon to the Lepidoptera) suggest a smaller number of ORs as compared to species belonging to more recent lineages of the Lepidoptera [73–75]. However, no such phylogenetic trend is clear for the ORs in Coleoptera [53]. Additionally, the polyphagous cerambycid *A. glabripennis* and the oligophagous chrysomelid *L. decemlineata* belong to the same superfamily (Chrysomeloidea), which is sister to the superfamily of the stenophagous *D. ponderosae* (Curculionoidea) [72]. Hence, restricting the comparison to these three relatively related species suggests an increasing number of chemosensory genes with a broader host range. Similarly, larger repertoires of ORs and GRs in the *Culex quinquefasciatus* mosquito is thought to be attributed to its broader host range (humans, livestock and birds) as compared to the more host-specific mosquitos *Anopheles gambiae* and *Aedes aegypti* [76]. Moreover, in drosophilids, the specialist *D. sechellia* is losing OR and GR genes at a faster pace than its generalist congeners *D. melanogaster* and *D. simulans* [77]*.* On the other hand, the sizes of the chemoreceptor gene repertoires are relatively conserved across sixteen species of *Anopheles* species irrespective of host range, suggesting that correlations between chemoreceptor repertoires and host range are not always evident, at least not among congeners [78].

### Evolutionary divergence and conservation of chemosensory genes

Larger gene-lineage expansions in *D. ponderosae* and *A. planipennis* are restricted to the three receptor gene families, and simple orthologous relationships among receptors were generally rare. In the present comparison with the polyphagous species, the positions in the trees of the expansions appear to be “arbitrary”, but may possibly reflect differences in ecological traits, such as feeding from conifers (*D. ponderosae*), angiosperms (*A. planipennis* and *A. glabripennis*), or stored products (*T. castaneum*). However, an analysis of the ORs from ten beetle genomes suggests that the distribution of ORs across the tree is at least partly dictated by the coleopteran phylogeny, with more similar distributions observed in closely compared to distantly related taxa [53]. This in turn implies that convergent evolution is an important driver for the function of ORs, since many compounds, such as green leaf volatiles, are detected by beetle species from a variety of families [64, 79, 80]. Similar to other bark beetles and weevils [66, 81, 82], the largest expansions of ORs in *D. ponderosae* are found within subfamily 7, followed by 5A and 1. Interestingly, *D. ponderosae* appears to have lost ORs from several subfamilies that are largely conserved across the Coleoptera. Especially noteworthy are subfamilies 3 and 5B, with the former housing relatively large proportions of the Apla-, Agla-, Tcas-, and LdecORs, and the latter containing one or a few ORs from most beetles investigated so far [53]. Functional data from ORs of these two subfamilies are needed to better understand whether or not the losses observed in *D. ponderosae* are related to its ecology. In contrast, *A. planipennis* has no ORs in subfamilies 7, 5A, or 1, and most of its ORs belong to subfamily 2B, which is highly reduced in *D. ponderosae*. However, a noteworthy common denominator in the two stenophagous wood-borers is their lack of ORs in subfamily 4, which is unique among the beetles investigated so far [53]. Whether this relates to ecological similarities, or if it is a coincidence remains unknown.

Conserved GRs for carbon dioxide were found in both stenophagous wood-borers, but GR1 was not recovered from the *A. planipennis* assembly or from the raw sequence data. While drosophilids lack GR2 and still detect carbon dioxide [22], it is unclear whether the apparent lack of GR1 means that *A. planipennis* has lost this ability, or if the *AplaGr1* gene simply was missed in the sequencing of *A. planipennis.* Both genomes contained genes for six putative sugar receptors (GR4–9) and one fructose receptor (GR10), which is fewer than in the two polyphagous species and might relate to their narrow host ranges. Reduction in candidate sugar receptor lineages was previously observed in the gall-midge *Mayetiola destructor* (Diptera), which is specialized on wheat and a few other grasses [52]. Similar to the ORs, putative bitter taste GRs were primarily distributed in species-specific lineage expansions at different positions in the phylogeny, with very few orthologuous relationships observed. The only orthologous clade was termed the “GR215” clade (following the orthologue from *T. castaneum*), and functional studies of these receptors are warranted to shed light on this conservation. Notably, *A. planipennis* is missing GRs from a major clade of our tree, suggesting a loss of GRs in this species, or that these GRs originated in more recent coleopteran lineages.

Although we identified fewer IRs in the stenophagous compared to the polyphagous species, the reduction was not as large as for the ORs and GRs. All members of the conserved olfactory antennal IRs [25] and members of the divergent IR100a clade were present in both *D. ponderosae* and *A. planipennis*. Duplications have occurred for *DponIr41a* and *AplaIr76b*, which is also the case in several species of Lepidoptera [51]. Furthermore, our analysis suggests the existence of orthologues of *DmelIr60a* in all beetle species. This orthologue is also conserved in moths and caddisflies, demonstrating a broad occurrence of this receptor in insects [73]. Surprisingly, the genome of *D. ponderosae* encoded more IR75 members (11) than any other beetle genome. IR75 paralogs have been implicated in olfaction and/or taste in moths based on expression patterns [51], and underlie responses to propionic acid in *D. melanogaster* [24]. Functional characterization will be necessary to confirm their putative olfactory roles in *D. ponderosae*.

We revealed four SNMPs in each of the three wood-boring species, which included two members in *A. planipennis* that are related to TcasSNMP3 [33]*.* The absence of SNMP3 in several beetle species suggests that its taxonomic occurrence is restricted. *T. castaneum* has a total of six SNMPs, and is only exceeded by *M. destructor* in terms of genomic content of SNMP genes [83]. While SNMP1 is important for pheromone responses in some species [31], the roles of different SNMP1 paralogues [84], SNMP2, and SNMP3 remain unknown, although transcripts of TcasSNMP3 are enriched in antennae and mouthparts, which suggests a putative role in chemosensation [33].

The OBP and CSP gene families are devoid of large species-specific radiations in *D. ponderosae* and *A. planipennis*, and the OBP family is especially reduced in both species. In contrast to the receptors, several orthologous clades with proteins from all four species were present in both gene families, suggesting that the evolutionary forces acting on these genes are different as compared to those acting on the receptor genes. The DponOBP family also exhibited alternative splicing and a “tetramer” gene form, neither of which have been previously reported from insects. Two genes (*DponObp6a/b* and *DponObp7a/b*) alternated a first exon that encoded the signal peptide, a hydrophobic motif that allows excretion of the protein from the cell. Previous transcriptome data indicate that *DponObp6* is antenna-specific, whereas *DponObp7* is expressed in a variety of tissues, but not in the antenna (see Additional file 3: Figure S1 in [66]). Hence, a speculative adaptive value of tissue-specific signal peptide composition may be to facilitate OBP excretion from cells that may differ in phospholipid membrane composition. The tetramer, DponOBP4, consists of a signal peptide followed by four consecutive exons, each of which is homologous to a member of the “Minus-C” subfamily of OBPs. Similar gene forms have been found previously, but only as pairs of exons (“dimers”) [85], including a Minus-C dimer in *L. decemlineata* [49]. While there are multiple evolutionary scenarios that could explain the origin of this unusual gene, the most parsimonious mechanism would be that the two “Minus-C” exons of an original dimer duplicated and were subsequently joined to the same gene as the parental exons. Alternatively, a duplicated complete dimer gene may have lost its short N-terminal exon, and the two main exons have then joined the upstream dimer. Unfortunately, our phylogenetic analysis did not conclusively demonstrate which scenario is the most plausible, although the comparatively high amino acid identity between exons 2 and 4 suggests a duplication event has been involved. The analysis also suggests that the sequences of the four exons have diverged significantly and that the duplication event hence must be ancient, which is supported by apparent orthologues in other curculionids. *DponObp4* is not expressed in the adult antennae (see Additional file 3: Figure S1 in [66]), which is similar to the dimer OBP in *D. melanogaster* [85], and suggests that OBP dimers and tetramers may not be involved in olfaction.

## Conclusions

Our genomic annotation of the chemosensory gene families in three species of wood-boring beetles includes a large number of novel sequences and significant improvements of original datasets obtained from transcriptomes. Hence, this study is an important contribution to the known chemosensory genes in the Coleoptera, facilitating evolutionary analysis. The results suggest a correlation between host range and numbers of chemosensory genes in essentially all chemosensory gene families (i.e., ORs, GRs, IRs, OBPs, and CSPs), with reductions in stenophagous beetle species largely explained by limited lineage expansions in most gene families (including both putatively functional genes and pseudogenes), entire lineage losses, and less alternative splicing of GR genes. The reductions in gene numbers may be further augmented in *A. planipennis* due to its well-developed visual system. Our phylogenetic analysis shows that orthology is generally rare among chemoreceptors from different beetle families, but we also reveal conservation of IR60a across beetles, and a conserved GR clade that we named the GR215 clade. In *D. ponderosae* we suggest alternative splicing of two of its OR genes, and we also provide the first evidence of alternative splicing among the OBP genes and a novel OBP Minus-C tetramer. Expansion of the IR75 clade in *D. ponderosae* is interesting in relation to its specialized ecology and destructive potential as a pest. This study provides an important platform for future functional characterization of chemoreceptors and binding proteins, which is a crucial step towards improved control of these economically devastating forest pests [4].

## Methods

### Annotation of chemosensory genes

Exhaustive tBLASTn searches against the genome assemblies of *D. ponderosae* (Curculionidae), *A. planipennis* (Buprestidae), and *A. glabripennis* (Cerambycidae) were conducted to identify chemosensory genes, using query sequences from *I. typographus*, *D. ponderosae*, *T. castaneum*, *A. glabripennis*, and *L. decemlineata* [33, 49, 54, 56, 66]. Annotation in *D. ponderosae* was conducted using primarily the published assembly of the female genome (accession APGL01000000) [86]. The current male assembly (APGK01000000) is more fragmented and was only used, when possible, to complete or improve those gene models that presented gaps in the female assembly. BLAST searches against the male assembly were also undertaken to ensure that no genes had been missed in the annotation of the female genome, which identified two OBPs exclusively found in the male. The chemosensory genes of *A. planipennis* were annotated from a public genome assembly (PRJNA230921) made available through the i5K project, and were used with permission. The CSP and SNMP genes from *A. glabripennis* were annotated using the published genome assembly (GCA_000390285.2) [56]. An *e*-value of cut-off at 10^− 1^ was used in the BLAST searches except for the putative bitter-taste GRs and divergent IRs where a cut-off at 3 (or occasionally 10) was used to ensure that divergent sequences were not missed. HMMER 3.1b1 (Eddy SR, Wheeler TJ, & HMMER development team, HMMER: biosequence analysis using profile hidden Markov models. http://hmmer.org/) with Pfam profiles PBP_GOBP (PF01395) and OS-D (PF03392) were also used to identify potential OBPs and CSPs from the NCBI REFSEQ sequences. The protein sequences of all identified genes were then blasted against each respective genome until the annotation of all novel hits was completed. Gene annotations and determination of exon/intron structure were performed manually using Geneious software package 7.1.9. (Biomatters Ltd., Auckland, New Zealand) or CLC Main Workbench 8.1 (QIAGEN Aarhus A/S, Denmark). Scaffold locations and additional annotation details for all genes are reported in Additional file 1: Table S1. Amino acid sequences for all annotations in this study are present in Additional file 4.

Following established nomenclature guidelines, two criteria were considered for the naming of the ORs, most GRs, and the ‘divergent’ IRs. First, genes in tandem arrays on scaffolds were given consecutive numbers. Secondly, preliminary trees were constructed to assign numbers to genes following their phylogenetic position, which for tandemly duplicated genes corresponded to their scaffold locations (for additional details see [53]). Hence, several of the previously identified genes were assigned new numbers in the present study (see Additional file 2: Table S2 for correspondence). Conserved GRs for carbon dioxide were named GR1-GR3 [according to 22], non-fructose sugar receptors GR4-GR9, and one putative fructose receptor in *D. ponderosae* and *A. planipennis* was named GR10 in both species. The naming scheme for the remaining, putative bitter-taste GRs, followed the two criteria described for the ORs with consecutive numbering starting at GR11. The consecutive numbering of the divergent IRs started at IR101 to avoid implying orthology to unrelated IRs in *D. melanogaster*. Conserved antennal IRs and all SNMPs were named according to their orthologous relationships with proteins in *D. melanogaster* and *T. castaneum* [23, 31, 33]. With the exception of IR75e, the other IR75 members did not show simple orthologous relationships across beetles, and they were given the consecutive letter suffixes “a-d” and “f-k”. The previously identified CSPs and OBPs in *D. ponderosae* and *A. planipennis* [66, 67] that were confirmed in the present study retained their original names, since no consensus in the nomenclature exists for these families. The CSPs in *A. glabripennis* annotated here were named consecutively according to scaffold positions. Exons of OR genes were named exon A, B, C, D, and E following ancestral splicing patterns [53], where the N-terminal A exon frequently is divided by one to several introns (in these instances, exons were named A1, A2, A3, …, A#X, according to [53]). No specific exon nomenclature was employed for the other gene families.

Models of chemosensory genes were frequently incomplete due to assembly gaps, large introns, or small and/or highly divergent terminal exons. Hence, we added suffixes to these gene names according to established protocols [87, 88]. Proteins with missing or truncated N-terminal, C-terminal, or internal exons were given the suffixes NTE, CTE, and INT, respectively (but partial models from *T. castaneum* retained their PAR suffix as previously reported). Gene models split across scaffolds were given a JOI suffix, which were confirmed by transcriptomic data when available [66, 69]. Models extended or corrected using raw genomic and transcriptomic read data were noted as FIX. A PSE suffix was added to models regarded as putative pseudogenes, including genes containing premature stop codon(s), frameshifts, missing start codon, exon(s), and/or splice sites. For models with more than one suffix, one-letter suffix abbreviations were used in relevant combinations (i.e., N, C, I, J, F, P). For several of the GRs, two ORs and two OBPs in *D. ponderosae*, we found indications of alternative splicing with mutually-exclusive N-terminal exons assembled consecutively with one or several seemingly shared C-terminal exons. Potential splice variants were named as e.g., GR#a, GR#b, GR#c etc. by convention [49]. Highly degenerated pseudogenes or fragments of putatively functional genes corresponding to single or a few short exons were discarded to improve alignments and phylogenetic analysis, and to avoid over-estimation of gene counts.

### Phylogenetic analyses

Multiple sequence alignments of protein sequences were performed using MAFFT v 7.017 [89], implemented in Geneious software package v7.1.9. The alignments were manually adjusted when necessary to correctly align some of the partial protein sequences. Uninformative regions were excised using trimAl v1.2 [90] with the following settings: similarity threshold 0, gap threshold 0.7, minimum 25% of conserved positions. The trimmed alignments of GR, IR, SNMP, OBP, and CSP proteins were used to construct trees using FastTree v2.1.10, at default settings [91]. Local branch support values were calculated using the Shimodaira-Hasegawa (SH) test implemented within FastTree. However, when constructing preliminary OR trees, FastTree performed inconsistently, with the tree topology affected by minor adjustments to the alignment and the inclusion/exclusion of individual ORs. Hence, we instead employed PhyML 3.0 [92] to perform phylogenetic analysis for this gene family, which also facilitated direct comparisons with the across-Coleoptera OR trees recently constructed using this method [53]. Prior to tree construction, PartitionFinder 2 [93] was used to select a model of gene evolution, with the best fit obtained for a JTT amino acid substitution matrix, gamma distributed rate variation, and empirical equilibrium amino acid frequencies (JTT + G + F). Branch support was calculated by aLRT (approximate Likelihood Ratio Test) SH-like likelihood ratios, implemented in PhyML. Trees were visualized and color coded in FigTree v1.4.3 [94], and graphically adjusted in Adobe Illustrator and Corel PaintShop Pro 2018 (Corel Corporation, Ottawa, Canada). Pseudogenes from *A. glabripennis* and *T. castaneum* were excluded from the phylogenetic analyses of all chemosensory gene families.

The OR sequences from *D. ponderosae* and *A. planipennis* were analyzed together with those from *A. glabripennis* [56] and *T. castaneum* (Tenebrionidae)*.* The OR set used for the latter species was an updated version of the original dataset [54], which included 22 revised gene models [33]. Partial OR sequences below 200 amino acids were excluded from the analysis, except for DponOR53INT (195 amino acids) in order to include all ORs from this study-species. To improve the legibility of the tree, the two massively expanded and species-specific lineages of TcasORs, originally classified as OR subfamilies 5 and 6 [54], were each represented by 10 TcasORs. The phylogenetic analysis of the GR family included the same species as the OR analysis. GR sequences from *A. glabripennis* were retrieved from McKenna et al. [56], and *T. castaneum* GRs from *Tribolium* genome sequencing consortium [55]. Due to the highly divergent nature of this receptor family, sequences below 250 amino acids were excluded to allow for a more robust phylogenetic analysis. The analysis of the IRs proceeded similarly, but also included the IRs from *L. decemlineata* (Chrysomelidae) [49] to facilitate inferences of orthologuous relationships across beetles. Sequences were derived from the same sources as described above, apart from the TcasIRs for which we used a revised dataset [49]. We also included the widely-conserved antennal IR orthologues from *D. melanogaster* (Diptera) [25] to support our naming of conserved IRs in the beetles and to infer across-order conservation. The SNMP analysis included the same species as the OR and GR trees, but also *D. melanogaster*. Sequences of SNMPs from *T. castaneum* were retrieved from Dippel et al. [33], and *D. melanogaster* SNMPs from GenBank (accession numbers: ABW70129.1 and NP_650953.1). Croquemort proteins, which are non-SNMP members of the CD36 family, from *D. melanogaster* and *T. castaneum* (accession numbers NP_787957.1 and XP_008192356.1), were included to root the tree. The OBP and CSP analyses included sequences from *D. ponderosae*, *A. planipennis*, *A. glabripennis*, and *T. castaneum.* OBPs from *A. glabripennis* were retrieved from Wang et al. [95], and OBPs and CSPs from *T. castaneum* from Dippel et al. [57].

## Additional files

Additional file 1: **Table S1.** Amino acid sequences and annotation details of the chemosensory genes identified in the genomes of *Anoplophora glabripennis*, *Agrilus planipennis*, and *Dendroctonus ponderosae.* Information on genomic location, gene structure, and protein length is presented alongside annotation notes where relevant. (XLSX 38 kb)

Additional file 2: **Table S2.** Gene name correspondence and revisions made to original models annotated from transcriptomes of *Dendroctonus ponderosae* and *Agrilus planipennis*. (XLSX 19 kb)

Additional file 3: **Figure S1.** *Left panel*: Unrooted phylogeny of select Minus-C odorant binding proteins (OBPs) to indicate the evolutionary relationships of the four main exons of DponOBP4. Included are OBPs from *Dendroctonus ponderosae* (Dpon, red), *Agrilus planipennis* (Apla, blue), *Anoplophora glabripennis* (Agla, green), and *Tribolium castaneum* (Tcas, orange). The tree is based on a trimmed MAFFT alignment, and constructed using FastTree. Numbers at nodes are local support values, calculated using the Shimodaira-Hasegawa (SH) test implemented in FastTree. Exact SH values are only shown if > 0.7, whereas SH values for all branches are indicated by the colored circles; support increases with the size and brightness of the circles. The sources of sequence data and explanation of protein suffixes are detailed in the Materials and Methods section. *Right panel*: Amino acid identity matrix of the four major exons of DponOBP4, calculated using Geneious software based on a MAFFT alignment. (JPG 719 kb)

Additional file 4: Translated amino acid sequences of all chemosensory genes annotated in the present study from *Anoplophora glabripennis*, *Agrilus planipennis*, and *Dendroctonus ponderosae*. (TXT 157 kb)

## Data Availability

All data generated or analyzed during this study are included in this published article and its supplementary information files. GenBank accession numbers for genome assemblies: *D. ponderosae* female APGL01000000; *D. ponderosae* male APGK01000000; *A. glabripennis* GCA_000390285.2; *A. planipennis* PRJNA230921. BLAST query sequences for *D. ponderosae* and *I. typographus* were retrieved from the Transcriptome Shotgun Assembly (TSA) database (accession numbers GABX00000000 and GACR00000000), and query sequences from *T. castaneum*, *A. glabripennis* and *L. decemlineata* were retrieved from previous publications (refs [33, 49, 54, 56, 57]).

## Abbreviations

Agla: *Anoplophora glabripennis*
Apla: *Agrilus planipennis*
CSP: Chemosensory protein
Dmel: *Drosophila melanogaster*
Dpon: *Dendroctonus ponderosae*
GR: Gustatory receptor
iGluR: Ionotropic glutamate receptor
IR: Ionotropic receptor
Ldec: *Leptinotarsa decemlineata*
OBP: Odorant binding protein
OR: Odorant receptor
OSN: Olfactory sensory neuron
SH: Shimodaira-Hasegawa
SNMP: Sensory neuron membrane protein
Tcas: *Tribolium castaneum*
