# Supplementary figures and images for: Genomic content of chemosensory genes correlates with host range in wood-boring beetles (Dendroctonus ponderosae, Agrilus planipennis, and Anoplophora glabripennis)
Source: BMC Genomics. 2019 Sep 2;20:690. doi: 10.1186/s12864-019-6054-x (PMC6720082; doi:10.1186/s12864-019-6054-x)

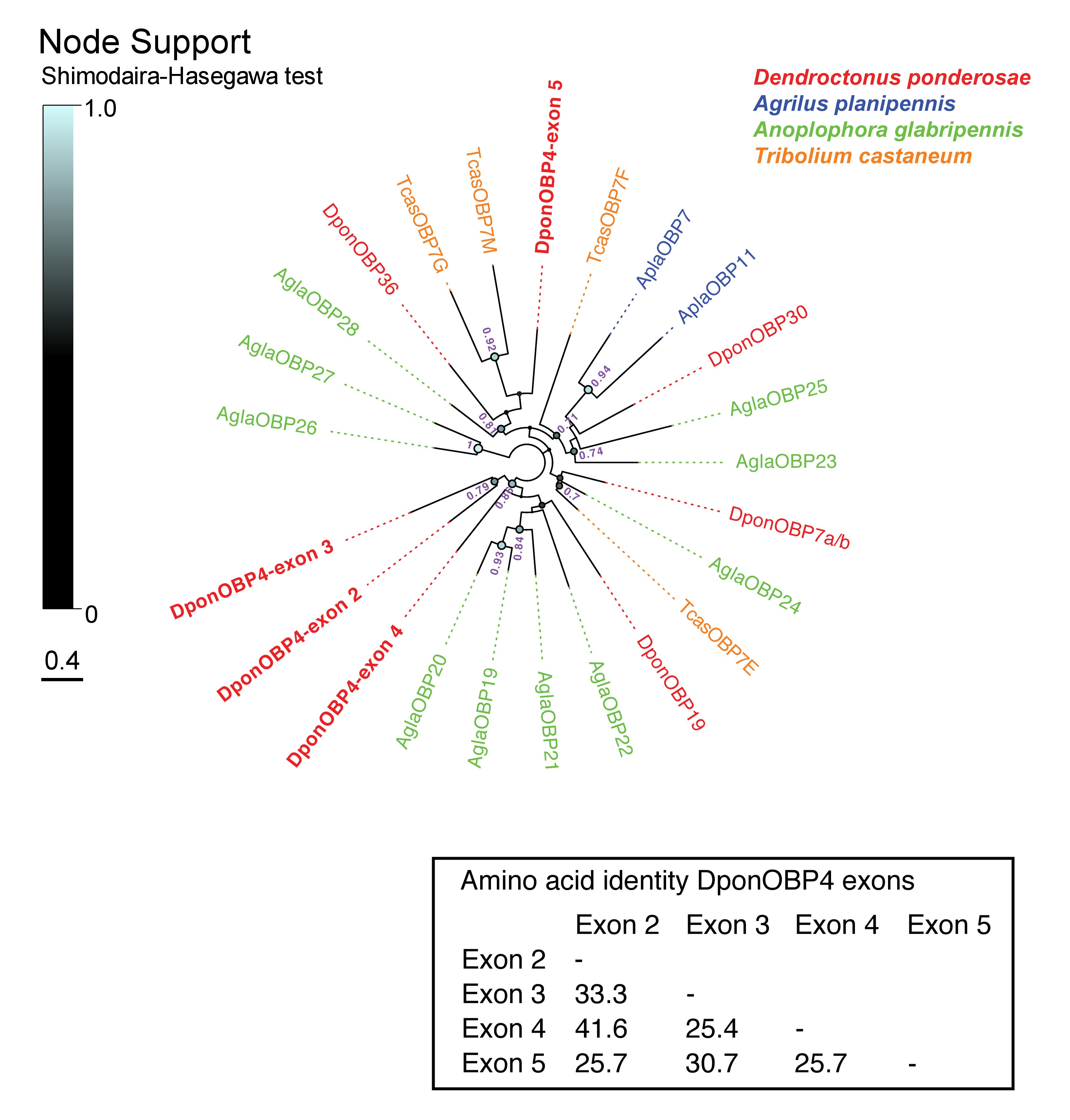

Supplement: Supplementary file 3 — Figure S1. Left panel: Unrooted phylogeny of select Minus-C odorant binding proteins (OBPs) to indicate the evolutionary relationships of the four main exons of DponOBP4. Included are OBPs from Dendroctonus ponderosae (Dpon, red), Agrilus planipennis (Apla, blue), Anoplophora glabripennis (Agla, green), and Tribolium castaneum (Tcas, orange). The tree is based on a trimmed MAFFT alignment, and constructed using FastTree. Numbers at nodes are local support values, calculated using the Shimodaira-Hasegawa (SH) test implemented in FastTree. Exact SH values are only shown if > 0.7, whereas SH values for all branches are indicated by the colored circles; support increases with the size and brightness of the circles. The sources of sequence data and explanation of protein suffixes are detailed in the Materials and Methods section. Right panel: Amino acid identity matrix of the four major exons of DponOBP4, calculated using Geneious software based on a MAFFT alignment. (JPG 719 kb) [file 12864_2019_6054_MOESM3_ESM.jpg]
